# Supplementary material for: Identifying the barriers and facilitators to culturally responsive HIV and PrEP screening for racial, ethnic, sexual, and gender minoritized patients: A scoping review protocol
Source: PLoS One. 2023 May 24;18(5):e0281173. doi: 10.1371/journal.pone.0281173 (PMC10208460; doi:10.1371/journal.pone.0281173)
Supplement: Peer review history

# Editor report: Decision Letter 0

**DOI:** 10.1371/journal.pone.0281173.r001

**Authors:** Alicia Chang

20 Mar 2023

Identifying the barriers and facilitators to culturally responsive HIV and PrEP screening for racial, ethnic, sexual, and gender minoritized patients: A scoping review protocol

PONE-D-23-00505

Dear Dr. Corr,

We’re pleased to inform you that your manuscript has been judged scientifically suitable for publication and will be formally accepted for publication once it meets all outstanding technical requirements.

Within one week, you’ll receive an e-mail detailing the required amendments. When these have been addressed, you’ll receive a formal acceptance letter and your manuscript will be scheduled for publication.

An invoice for payment will follow shortly after the formal acceptance. To ensure an efficient process, please log into Editorial Manager at http://www.editorialmanager.com/pone/, click the 'Update My Information' link at the top of the page, and double check that your user information is up-to-date. If you have any billing related questions, please contact our Author Billing department directly at authorbilling@plos.org.

If your institution or institutions have a press office, please notify them about your upcoming paper to help maximize its impact. If they’ll be preparing press materials, please inform our press team as soon as possible -- no later than 48 hours after receiving the formal acceptance. Your manuscript will remain under strict press embargo until 2 pm Eastern Time on the date of publication. For more information, please contact onepress@plos.org.

Kind regards,

Alicia Chang, M.D., M.S.

Academic Editor

PLOS ONE

Additional Editor Comments (optional):

1. Please ensure that you have specified (1) whether consent was informed and (2) what type you obtained (for instance, written or verbal, and if verbal, how it was documented and witnessed). If your study included minors, state whether you obtained consent from parents or guardians. If the need for consent was waived by the ethics committee, please include this information

2. Thank you for stating the following financial disclosure:

"The authors have declared that no competing interests exist.".

e)        Please provide an amended Funding Statement that declares *all* the funding or sources of support received during this specific study (whether external or internal to your organization) as detailed online in our guide for authors at http://journals.plos.org/plosone/s/submit-now. 

f)        Please state what role the funders took in the study.  If any authors received a salary from any of your funders, please state which authors and which funder. If the funders had no role, please state: "The funders had no role in study design, data collection and analysis, decision to publish, or preparation of the manuscript."

Please send your amended statements by return email; we will change the online submission form on your behalf.

This is a well written article describing a study protocol to gather broad data on barriers to as well as promoters of PrEP eligibility screening among historically marginalized populations.

The manuscript presents a thorough background and potential impact and details the methodology well.

The authors take an interesting approach proposing a scoping review to synthesize existing scientific literature. As a less familiar review method, it's highly worthwhile to present examples of study protocols in the literature, and this manuscript serves that purpose well.

​

# Editor report: Acceptance letter

**DOI:** 10.1371/journal.pone.0281173.r002

**Authors:** Alicia Chang

16 May 2023

PONE-D-23-00505 

Identifying the barriers and facilitators to culturally responsive HIV and PrEP screening for racial, ethnic, sexual, and gender minoritized patients: A scoping review protocol 

Dear Dr. Corr:

I'm pleased to inform you that your manuscript has been deemed suitable for publication in PLOS ONE. Congratulations! Your manuscript is now with our production department. 

If your institution or institutions have a press office, please let them know about your upcoming paper now to help maximize its impact. If they'll be preparing press materials, please inform our press team within the next 48 hours. Your manuscript will remain under strict press embargo until 2 pm Eastern Time on the date of publication. For more information please contact onepress@plos.org.

If we can help with anything else, please email us at plosone@plos.org. 

Thank you for submitting your work to PLOS ONE and supporting open access. 

Kind regards, 

PLOS ONE Editorial Office Staff

on behalf of

Dr. Alicia Chang 

Academic Editor

PLOS ONE
